# Editorial: Urban green spaces and human health

**DOI:** 10.3389/fpubh.2024.1404452

**Published:** 2024-06-03

**Authors:** Yuan Li, Hongxiao Liu, Diogo Guedes Vidal, Abdullah Akpınar, Ding Li

**Affiliations:** ^1^Northwest Land and Resource Research Center, Shaanxi Normal University, Xi'an, Shaanxi, China; ^2^CAS Engineering Laboratory for Vegetation Ecosystem Restoration on Islands and Coastal Zones, Key Laboratory of Vegetation Restoration and Management of Degraded Ecosystems, South China Botanical Garden, Chinese Academy of Sciences, Guangzhou, China; ^3^Department of Life Sciences, Center for Functional Ecology, Faculty of Sciences and Technology, University of Coimbra, Coimbra, Portugal; ^4^Department of Social Sciences and Management, Universidade Aberta, Lisbon, Portugal; ^5^Department of Landscape Architecture, Faculty of Agriculture, Aydın Adnan Menderes University, Aydın, Türkiye; ^6^Institute of Development Studies, Southwestern University of Finance and Economics, Chengdu, China

**Keywords:** green space, disease, mental health, public health, healthy aging

An essential component of fostering sustainable and regenerative urban development lies in elevating collective wellbeing standards (1, 2). The United Nations officially adopted the 2030 Agenda for Sustainable Development in 2015, outlining 17 Sustainable Development Goals (SDGs) aimed at eradicating poverty, safeguarding the environment, and enhancing the quality of life for all individuals globally by 2030. Urban Green Spaces (UGSs) play a pivotal role in influencing living conditions and public health, directly contributing to several SDGs such as promoting good health and wellbeing (SDG 3), ensuring access to clean water and sanitation (SDG 6), fostering industry, innovation, and infrastructure (SDG 9), and advancing sustainable cities and communities (SDG 11).

The principle of One Health assumes paramount significance by acknowledging the intricate interplay among humans, animals, and the environment as a critical determinant of public health and wellbeing (3). This recognition underscores the necessity for interdisciplinary collaborations to cultivate a comprehensive understanding and implement effective measures against public health challenges (4). However, there are certain drawbacks to the “One Health” strategy. The focus primarily on zoonotic illnesses may inadvertently obscure other environmental health concerns, such as pollution and non-communicable diseases (5). Moreover, there is a chance that this concept may unduly emphasize the features of biological health, thereby ignoring the social and cultural dimensions that have a significant impact on wellbeing. However, the “Eco-Health” approach provides a more comprehensive understanding of health, considering environmental, social, and economic factors that affect health outcomes (6). This strategy emphasizes how critical it is to involve and engage the community in the identification and resolution of health issues (7).

Research has consistently demonstrated the manifold benefits of UGSs on the mental, physical, and social wellbeing of residents (8). Various studies have established correlations between green spaces (e.g., quality, spatial distribution, biodiversity, proximity to residential areas) and health outcomes, encompassing birth weight, adult excessive body weight and obesity, mental and cardiovascular health, and overall mortality rates (9, 10). These findings underscore the indispensable role of green spaces in enhancing the daily lives of urban dwellers. Despite the wealth of evidence showcasing the interconnectedness of human health and UGSs, there remains a notable research gap in this domain.

The Research Topic, “*Urban green spaces and human health*” featured in the “Environmental Health and Exposome” section of Frontiers in Public Health, aims to comprehensively explore the impact of UGSs on human health. The focus is broad yet specific, encompassing various key areas including (a) UGSs for enhancing public health; (b) Links between UGSs and disease; (c) Influence of UGS quality on mental health; (d) Promotion of sustainable and regenerative urban development through strategic UGS planning; (e) Case studies illustrating the efficacy of greenspace interventions in fostering healthy communities and cities; (f) Examination of the relationship between UGSs and marginalized communities; (g) Utilization of big data mining techniques to enhance the quality of green spaces; (h) Qualitative approaches to understanding the effects of UGSs on human health; (i) Identification of challenges and opportunities associated with the intersection of UGSs and public health; (j) Evaluation of urban living conditions for older adults in the 21st century; and (k) Cross-country disparities in healthy aging within urban settings.

Under this Reaearch Topic, 18 articles have been successfully published with relevant findings contributing to the advancement of research on the impact of UGSs on human health. Deng et al. conducted a meta-analysis on the role of greenways in promoting physical activity, emphasizing the need to recognize greenways as an effective public health intervention. Cao et al.'s study investigated the impact of different environmental conditions on public physiological and psychological health in UGSs, highlighting the significant influence of weather on the restorative potential of these spaces. Li H. et al. delved into the correlation between green recreational activities, residential green spaces, and mental health, reinforcing established “green space-health” frameworks and underscoring the importance of leisure physical activity in boosting mental wellbeing. Zheng et al. laid the groundwork for comprehending residents' spatial experiences and behavioral requirements, conducting a scientific evaluation of UGS quality, and optimizing the structure of community green spaces. Li Q. et al. shed light on the substantial influence of UGSs on the wellbeing of middle-aged and older adults. Zhang T. et al. scrutinized the perceptions of health risks associated with hot weather and the cooling benefits of UGSs, stressing the role of green spaces and water in alleviating urban heat threats and residents' health risk perceptions. Guo et al. identified the essential landscape elements required for hospital rehabilitation spaces through an empirical study of 10 small hospitals. Zhang C. et al.'s research focused on the urban park system for public health, advocating for the optimal development strategy of urban parks at both macro and micro levels to promote sustainable urban public health. Kolster et al. conducted a controlled trial on guided nature walks or group exercises for health promotion in primary care, demonstrating the benefits of nature-based interventions, even in green surroundings, for improving health. Mohr-Stockinger et al.'s study aimed to optimize biodiversity-friendly residential greening to promote health. They highlighted that neighbors are already highly motivated to actively participate in creating locally adapted solutions and taking responsibility for optimizing residential green spaces for health promotion. Fu et al. explored the constraints of community greenways for physical activity using a structural equation model. Their findings provide insights for enhancing people's willingness to utilize greenways for physical activity and offer a theoretical basis for the healthy design and transformation of community greenway spaces. Yang et al. focused on evaluating the quality of life and spatial correlations in impoverished areas of Guizhou Province. They found that while the overall quality of life in all impoverished districts and counties of Guizhou Province has improved, significant disparities in quality of life between the eastern and western regions of the province persist. Xia studied the impact of green spaces on residents' wellbeing. They emphasized that only through an understanding of the relationship between cultural heritage and green development can a virtuous cycle of development be created, thereby promoting the continuous development of a unique and historically significant urban area. Lak et al. examined the impact of older adult-friendly public open spaces in urban impoverished communities on the health of older adults. They highlighted that the personal aspect, socio-demographic status, place preferences, and environmental processes collectively influence the health of older adults. Rose and Riley suggested that key concepts of the five ways to wellbeing can serve as a framework for zoos to engage more effectively with their human audiences. Li J. et al. investigated tourists' perceptions of historic districts, landscape perception, and place attachment. They found that landscape perception significantly influences perceived restoration, with indirect effects through place dependence and identity, as well as a direct impact of landscape perception. Yan et al. studied the psychological health recovery of older adult individuals in parks during different seasons. In winter, perceived environment assessment was not a direct antecedent of restorative effects, with moderate and vigorous physical activity feedback serving as important mediating factors. In seasons other than winter, low physical activity feedback played crucial mediating roles.

Future research in the field of *Urban green spaces and human health* should focus on exploring the differential health benefits of UGSs across various population groups, considering factors such as age, socioeconomic status, and cultural backgrounds. Additionally, there should be a growing emphasis on how urban planning and design can maximize the positive impact of green spaces on mental health, as well as strategies to Protect and promote the sustainability of green spaces in urban development, ensuring better health and wellbeing for future urban residents.

Thank all authors who contributed to this research theme, and we invite readers to explore the excellent articles in this compilation.

## Author contributions

YL: Conceptualization, Funding acquisition, Writing – original draft, Writing – review & editing. HL: Funding acquisition, Writing – review & editing. DV: Funding acquisition, Writing – review & editing. AA: Writing – review & editing. DL: Writing – review & editing.

## References

[1] Blanco E Raskin K Clergeau P. Towards regenerative neighbourhoods: an international survey on urban strategies promoting the production of ecosystem services. Sustain Cities Soc. (2022) 80:103784. 10.1016/j.scs.2022.103784

[2] Camrass K. Urban regenerative thinking and practice: a systematic literature review. Build Res Inf. (2022) 50:339–50. 10.1080/09613218.2021.1922266

[3] Vidal DG Oliveira GM Pontes M Maia RL Ferraz MP. Chapter 6 - The influence of social and economic environment on health. In:Prata JC Ribeiro AI Rocha-Santo T, editors. One Health. Integrated Approach to 21st Century Challenges to Health. Cambridge, MA: Academic Press (2022), p. 205–29. 10.1016/B978-0-12-822794-7.00005-8

[4] Humensky JL Abedin Z Muhammad K McClave M Torres T DiMaria ES. Promoting interdisciplinary research to respond to public health crises: the response of the Columbia University CTSA to the opioid crisis. J Clin Transl Sci. (2020) 4:22–7. 10.1017/cts.2019.426 32257407 PMC7103466

[5] Johnson I Hansen A Bi P. The challenges of implementing an integrated one health surveillance system in Australia. Zoonoses Public Health. (2018) 65:E229–36. 10.1111/zph.12433 29226606 PMC7165821

[6] Harrison S Kivuti-Bitok L Macmillan A Priest P. Ecohealth and one health: a theory-focused review in response to calls for convergence. Environ Int. (2019) 132:105058. 10.1016/j.envint.2019.105058 31473414

[7] Lencastre M Saraiva R Calheiros J Vidal DG Barroso EP Campelo A. Composing worlds: a portuguese transdisciplinary network in humanities, health and well-being. Societies. (2023) 13:97. 10.3390/soc13040097

[8] Vidal DG Dias RC Oliveira GM Dinis MAP Fernandes CO Filho WL. A review on the cultural ecosystem services provision of urban green spaces: perception, use and health benefits. In:Leal Filho W Vidal DG Dinis MAP Dias RC, editors. Sustainable Policies and Practices in Energy, Environment and Health Research. Cham: Springer (2022), p. 287–331 10.1007/978-3-030-86304-3_18

[9] Liu HX Ren H Remme RP Nong HF Sui CH. The effect of urban nature exposure on mental health-a case study of Guangzhou. J Clean Prod. (2021) 304:127100. 10.1016/j.jclepro.2021.12710032543435

[10] Liu HX Li F Li JY Zhang YY. The relationships between urban parks, residents' physical activity, and mental health benefits: a case study from Beijing, China. J Environ Manage. (2017) 190:223–30. 10.1016/j.jenvman.2016.12.058 28056355

